# Utilization and uptake of the UpToDate clinical decision support tool at the Makerere University College of Health Sciences (MakCHS), Uganda

**DOI:** 10.4314/ahs.v21i2.52

**Published:** 2021-06

**Authors:** Alison Annet Kinengyere, Julie Rosenberg, Olivia Pickard, Moses Kamya

**Affiliations:** 1 Makerere University College of Health Sciences Library; 2 Ariadne Labs, Brigham and Women's Hospital; 3 Makerere University College of Health Sciences

**Keywords:** UpToDate clinical decision support tool, Makerere University College of Health Sciences, Uganda

## Abstract

**Background:**

The use of point-of-care, evidence-based tools is becoming increasingly popular. They can provide easy-touse, high-quality information which is regularly updated and has been shown to improve clinical outcomes. Integrating such tools into clinical practice is an important component of improving the quality of health care. However, because such tools are rarely used in resource-limited settings, there is limited research on uptake especially among medical students.

**Objective:**

This paper explores the uptake of one such tool, Up-To-Date, when provided free of cost at a medical school in Africa.

**Methods:**

In partnership with the Better Evidence at Ariadne Labs free access to UpToDate was granted through the MakCHS IP address. On-site librarians facilitated training sessions and spread awareness of the tool. Usage data was aggregated, based on log ins and content views, presented and analyzed using Excel tables and graphs.

**Results:**

The data shows evidence of meaningful usage, with 43,043 log ins and 15,591 registrations between August 2019 and August 2020. The most common topics viewed were in obstetrics and gynecology, pediatrics, drug information, and infectious diseases. Access occurred mainly through the mobile phone app.

**Conclusion:**

Findings show usage by various user categories, but with inconsistent uptake and low usage. Librarians can draw upon these results to encourage institutions to support uptake of point-of-care tools in clinical practice.

## Background

Digital point-of-care tools can provide easy-to-use, high-quality information that is regularly updated in line with the science and have been shown to improve diagnostic accuracy and promote quality, efficient care^1^. The use of such tools is becoming increasingly popular throughout the world. A majority of the increased use is happening in North America, though Asia Pacific is anticipated to be the fastest growing region in adopting these tools 2. An American Medical Association survey showed that 57% of physicians use or plan to use a digital clinical support tool in their work 3. The clinical decision support resource UpToDate is used in over 190 countries and by 90% of US academic medical centers 4. Integrating these tools into practice, particularly in sub-Saharan Africa, is an important component of improving the overall quality of healthcare. However, significant gaps exist in the implementation of these tools into healthcare providers' routine practice in low-resource settings 5. Though medical students in the US are likely to be introduced to these digital tools early in their career 6, the use of digital tools has not gained the same momentum in sub-Saharan African medical education 7. Research conducted by the authors of this study at the University of Rwanda suggests that early introduction of an evidence-based tool to medical students leads to habit formation and use of the tool in later clinical practice 8. However, there has been limited research on this topic, likely due to limited access to such tools whose cost can be prohibitive. Sub-Saharan African medical schools have faced insufficient access to digital clinical resources, with medical schools rating their technological resources somewhat to severely inadequate on average 9.

## About UpToDate

UpToDate is a clinical decision support resource that can be used on or offline and provides evidence-based information for medical doctors. It can be accessed on digital devices such as computers, tablets, or mobile smart phones in hospitals, clinics, or homes. The tool is authored by 7,100 physicians who continuously synthesize the most recent medical information across specialties into evidence-based recommendations that can support point-of-care decisions. UpToDate is used in over 190 countries and by 90% of US academic medical centers^4^.

Recent research demonstrates that the use of UpToDate was associated with improved quality of care, shorter lengths of stay, and lower mortality rates over a three-year period^1^. UpToDate has been shown to answer clinical questions effectively, with one study citing an 86% answer retrieval rate for UpToDate 10. In medical education, UpToDate is reported to be a highly effective resource for learning 11 and is preferred by early career doctors 12.

## UpToDate Content

The tool covers a range of information areas and tools, including: topic updates by specialty, clinical calculators, drug interaction checkers, and search functionality (by disease name, symptom, lab abnormality, procedure, or drug) with filter options (adult, pediatric, or patient graphics). While users can search UpToDate in many languages, the content is available only in English. With the emergence of the COVID-19 pandemic, UpToDate has added new open-access information covering clinical topics, questions, patient education and society guidelines. The pace of discovery and science related to the COVID-19 pandemic as well as the spread of misinformation, coupled with shortages of health workers have only hastened the need for clinicians to have evidence-based and trusted resources to which they can turn for clinical information.

## Accessing UpToDate

UpToDate is a commercial product available for purchase. Better Evidence, a group at Ariadne Labs – a joint center for health systems innovations at Harvard T.H. Chan School of Public Health and Brigham and Women's Hospital – works to facilitate access to evidence-based clinical resources to health providers serving vulnerable populations who couldn't otherwise afford them. A pilot study run by Better Evidence demonstrated the utility of Up-To-Date among medical students at University of Rwanda^8^, the group began facilitating donated institutional licenses to medical schools across Africa as part of the Better Evidence for training program. Access was granted to MakCHS in 2019, with plans to add new schools annually.

With an institutional license, users can access UpToDate (www.uptodate.com) on the institutional local area network (LAN) and register for an individual account that will allow them to access UpToDate outside the LAN and download the content for use offline. The ability to use the tool offline is a valuable feature in developing countries.

## About the study site (MakCHS)

Established in 1922 as a technical school, Makerere University is one of the oldest and most prestigious English Universities in Africa. The college soon began offering various other courses in medical care, agriculture, veterinary sciences and teacher training. It expanded over the years to become a center for higher education in East Africa in 1935 13.

On July 1, 1970, Makerere became an independent national university of the Republic of Uganda, offering undergraduate and postgraduate courses. Makerere University offers not only day but also evening and external study programmes to a student body of about 35,000 undergraduates and 3,000 postgraduates (both Ugandan and foreign).

The university transitioned from the faculty-based to the collegiate system in 2011. As of July 2014, it includes 10 constituent colleges including the School of Law, all operating as semi-autonomous units 13.

Makerere University College of Health Sciences was transformed from a Faculty of Medicine into a College in 2013. It is comprised of 4 schools (Medicine, Biomedical Sciences, Public Health and Health Sciences) and 27 departments, with a total population of 3,018 students, 249 academic staff, who double as lecturers and health workers, mainly stationed at the Mulago National Referral and Teaching Hospital. It is this population, together with other health researchers who are affiliated to the College that utilizes the UpToDate subscription facilitated by Better Evidence to support clinical practice 14.

## Objective

This paper explores the uptake of UpToDate among medical students and faculty at Makerere University College of Health Sciences.

## Methodology

After being approved for participation in the Better Evidence for training program, MakCHS entered into a contract with UpToDate. UpToDate established access to the product through the LAN at the university and its affiliated training facilities in August 2019. Better Evidence team members aided librarians, students, and faculty at MakCHS in learning how to access, register for, and use the tool. The MakCHS librarians then communicated about the tool and built awareness of the tool in their respective institutions through training sessions on campus and messaging.

UpToDate tracked aggregate usage and searches by registered, logged-in users or users on campus and shared this data with the school through the Better Evidence program every two months. Usage of the open-access information on COVID-19 was not tracked if users were offsite and not logged in. No institutional review board approvals were needed given the nature of this work and the aggregate nature of the data that did not allow any individuals to be identified.

The data presented here captured usage information from August 2019 to August 2020 on the following:

- Trending topics sorted by most views—topics that increased in popularity from the prior two month period
- Methods used to access the topics—whether users accessed UpToDate via the website on a computer or via the UpToDate app on their smart phones, tablets or iPads
- Roles of the users that accessed UpToDate
- Top five medical topics by views —the most frequently visited topic cards during each two-month period
- Top five medical specialties viewed – what specialties the topics viewed fall into
- Total usage by month—how many searches were done

### Data analysis

We used Excel to organize the data into tables and graphs to enable us to look at the trends and usage patterns over time. We then grouped the relevant data based on trending topics, access methods, user roles, topic specialties by views, medical topics by views and total usage of the tool by month. Reporting periods are labeled by when the report was received. UpToDate provided reports every two months.

## Results

### Trending Topics

Table 1 shows the trending topics sorted by increase in views for each two-month reporting period. More recently trending topics have been related to one another. In general, trends show the wide array of topics viewed and how they can change from month to month.

**Table 1:** Trending Topics

Month of report	Topic viewed
**Aug-20**	Approach to the adult with acute diarrhea in resource-rich settings
	Preeclampsia: Clinical features and diagnosis
	Spontaneous abortion: management
**Jun-20**	Convulsive status epilepticus in adults: Treatment and prognosis
	Convulsive status epilepticus in adults: Classification, clinical features, and diagnosis
	Acute liver in children: Management, complications, and outcomes
**Apr-20**	Valvar lesions: differential diagnosis based on morphology
	Biliary atresia
	Congenital syphilis: clinical features and diagnosis
**Feb-20**	Epidemiology, clinical presentation, and diagnostic evaluation of para pneumonic effusion and empyema in adults
	Achalasia: Pathogenesis, clinical manifestations, and diagnosis
	Definition, classification, etiology, and pathophysiology of shock in adults
**Dec-19**	Treatment of pheocromocytoma in adults
	Acute complicated urinary tract infection (including pyelonephritis) in adults
	Management of locally advanced cervical cancer
**Oct-19**	Overview of hypertension in adults
	Invasive cervical cancer: Epidemiology, risk factors, clinical manifestations, and diagnosis
	Anemia in pregnancy

### Access Methods

**Figure 1:**
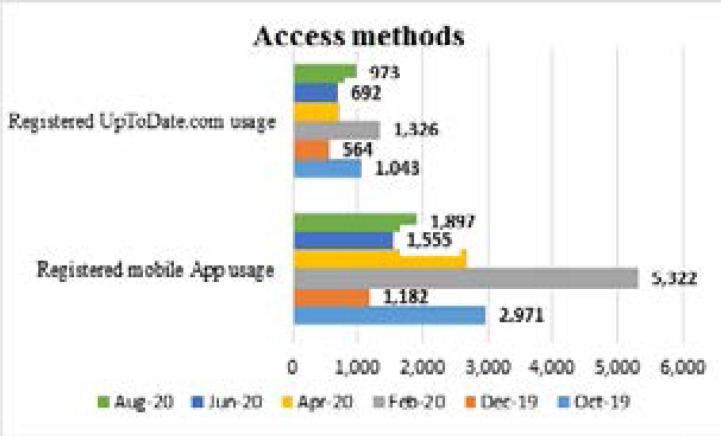
MakCHS: Access methods-Oct and Dec 2019, Feb -Aug 2020

Figure 1 shows the number of times the tool was accessed by registered users via the website or the app for each two-month period being reported in the months of October and December 2019, and February, April, June, and August 2020.

Usage via the appwas consistently about two to three times more common than usage via the website but both forms of usage followed approximately the same trends. The February 2020 report showed the most usage via both the web (1,326) and the app (5,322) while the December 2019 report showed the lowest usage of any two-month period.

### User roles

Table 2 shows the roles of the users who accessed UpToDate by reporting period. People with a range of roles registered for and accessed the tool. There were consistently a higher number of medical students and residents accessing the tool compared to physicians and other user types. February and April reporting periods showed the highest numbers of users accessing the tool across the top three roles, i.e., medical students, residents, and physicians. Physician assistants used the tool much more in the December 2019 reporting period than during any other reporting period.

**Table 2:** MakCHS: User roles that accessed the topics in Oct 2019, Dec 2019, Feb 2020 and Apr 2020

User role	Oct-19	Dec-19	Feb-20	Apr-20	June-20	Aug-20	Total users
Medical student	133	64	133	132	48	61	**571**
Resident	46	44	52	52	37	50	**281**
Physician	22	18	27	27	23	27	**121**
Other	5	0	1	2	-	2	**10**
Medical Librarian	1	1	0	0	-	2	**2**
Nurse	0	1	1	1	1	1	**5**
Pharmacist	1	1	2	2	1	1	**8**
Physician assistant	1	21	1	1	1	-	**25**
Nurse practitioner	0	0	1	1	1	1	**4**
TOTAL	209	150	218	218	112	145	1,027

Source: Better Evidence

### Medical specialties by views

The topics viewed fall into different specialty areas. The top five topic specialties viewed from October 2019 to August 2020 are shown in Table 3.

**Table 3:** MakCHS: Up-To-Date users by specialty during the months of Oct. 2019, Dec. 2019, Feb. 2020, Apr. 2020 and Jun. 2020

Top Five Topic Specialties Viewed	Oct-19	Dec-19	Feb-20	Apr-20	Jun20	Aug-20	TOTAL
Pediatrics	565	171	995	585	352	373	**3,041**
Obstetrics, gynecology & women's health	493	294	762	494	139	399	**2,581**
Infectious diseases	208	153	607	294	135	250	**1,647**
Drug information	-	223	-	297	172	360	**1,052**
Gastroenterology and Herpetology	-	-	518	-	121	-	**639**
	1,266	841	2,882	1,670	919	1,382	**8,960**

Source: Better Evidence

Pediatrics, obstetrics and gynecology & women's health registered the most topic hits over the course of the year, while gastroenterology and herpetology received a lot of views during two reporting periods.

### Medical topics by views

The top five most viewed medical topics by reporting period are shown in Table 4. The pattern suggests diverse usage. In some reporting periods, there are clear connections between the medical topics viewed, while in other periods there is quite a range of views. In general, the number of views for each specific topic is relatively low compared to the total number of views of all topics.

**Table 4:** Top Five Medical Topics by views

**Oct-19**	Overview of the clinical presentation and diagnosis of acute lymphoblastic leukemia/lymphoma in children (31) Pelvic organ prolapse in women: Diagnostic evaluation (29) Pelvic organ prolapse in women: Epidemiology, risk factors, clinical manifestations, and management (27) Normal and abnormal labor progression (26) Presentation, diagnosis, and staging of Wilms tumor (22)
**Dec-19**	Paragangliomas: Epidemiology, clinical presentation, diagnosis, and histology (15) Spinal anesthesia: Technique (15) Shoulder dystocia: Intrapartum diagnosis, management, and outcome (14) Peptic ulcer disease: Epidemiology, etiology, and pathogenesis (13) Intrapartum fetal heart rate assessment (11)
**Feb-20**	Traumatic brain injury: Epidemiology, classification, and pathophysiology (51) Hypospadias: Pathogenesis, diagnosis, and evaluation (45) Overview of the clinical manifestations of sickle cell disease (43) Uterine leiomyoma's (fibroids): Epidemiology, clinical features, diagnosis, and natural history (34) Approach to the patient with abnormal liver biochemical and function tests (30)
**Apr-20**	Vulvar lesions: Differential diagnosis based on morphology (27) Overview of the clinical manifestations of sickle cell disease (25) Neonatal necrotizing enterocolitis: Clinical features and diagnosis (23) Coronavirus disease 2019 (COVID-19): Epidemiology, virology, clinical features, diagnosis, and prevention (22) Management of acute moderate and severe traumatic brain injury (20)
**June-20**	Acute liver failure in children: Etiology and evaluation (29) Causes of cholestasis in neonates and young infants (27) Clinical manifestations and diagnosis of hepatitis B virus infection in children and adolescents (22) Convulsive status epilepticus in adults: Classification, clinical features, and diagnosis 22 Cervical cancer in pregnancy (19)
**Aug-20**	Uterine fibroids (leiomyoma's): Treatment overview (25) Antiphospholipid syndrome: Pregnancy implications and management in pregnant women (20) Infants with fetal (intrauterine) growth restriction (16) Cervical cancer in pregnancy (15) Overview of antepartum fetal surveillance (15)

Source: Better Evidence

### Total usage by month

**Figure 2:**
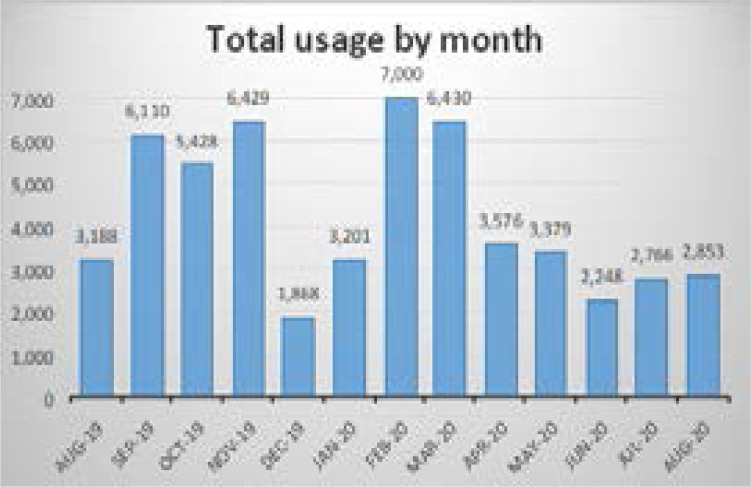
Total usage by month: Aug 2019-Aug 2020

Total usage of the tool by month is shown in Figure 2. Between August 2019 and September 2019, usage increased by nearly 90%. December 2019 saw the lowest usage while February 2020 saw the highest.

## Discussion

The discussion of findings is presented according to the subheadings discussed in the results section:

Access methods data shows that users primarily accessed UpToDate on mobile devices rather than computers. This suggests that users are able and willing to access the digital tool while on the go and not only in a static location. This is promising given the relevance of the tool for clinical care use at the patient bedside where there are often no computers. This also suggests that people are willing to use their own personal devices for learning and consulting the evidence given the school does not provide mobile devices. Furthermore, hospitals in Uganda, where the students and residents do ward rounds do not necessarily have internet connection, so the fact that use occurred on mobile devices suggests that users may have been able to download the UpToDate content and use the offline feature of the tool. The ability to use the tool offline is likely make the use of UpToDate on mobile devices more appealing and could be an important feature when considering use of digital tools in limited-resource settings.

User role data is promising and it shows those in their training years at the institution used Up-To-Date most. This finding is encouraging as these are critical years for establishing habits for practice for the years ahead. It is important to note however that the denominator for the various groups users groups varies, which may explain why some user groups appear to use the tool less. For example, the college has only three librarians compared to 2,700 students.

The beginning of the semester is characterized by exams and tests, and that is the time when lectures are gaining momentum. Students are therefore usually busy and this could have affected usage. The month of March 2020 was further affected by the lockdown and closure of institutions of higher education in Uganda due to the COVID 19 pandemic. This, coupled with the beginning of semester schedules, could have further affected usage.

Medical specialties and topics by views data shows that pediatrics, obstetrics and gynecology, infectious diseases, drug information, and gastroenterology and herpetology registered many and continuous viewers throughout the period indicated. While the increased views of the infectious diseases specialty during the month of February 2020 could be attributed to the COVID-19 pandemic, the various topic specialties viewed suggest just how one point-of-care tool can be used in a variety of situations that benefit various categories of users. The data also suggest that these few specialties are especially keen on using information that is up to date to facilitate the application of evidence-based medicine in daily clinical practice. The ability to access COVID-19 information without logging in also may have impacted the data on topics and specialties viewed.

Total usage by month data variation can mostly be explained by outside situational factors. For example, MakCHS received access to Up-To-Date in July 2019. Therefore, in August, most potential users were just becoming aware of the availability of the tool, and trainings were just starting. After being introduced to the tool, users started accessing it more. Between July and September, usage increased substantially. Trainings were conducted weekly during this period, suggesting a clear impact between training and usage. Usage then clearly decreased during the holiday break but came back up once classes resumed. In March 2020, MakCHS went into lockdown due to the COVID-19 pandemic and as of August 2020, the students were still under lockdown, although remote work had been eased for a few members of staff. The big difference between usage in February and June 2020 was therefore likely a result of the lockdown and closure of the university, due to the COVID-19 pandemic. There were no trainings or awareness campaigns creation during that time, and students were likely to have less internet access while remote as well.

It is probable that, had it not been for the lockdown due to the COVID-19 pandemic at the end of March, usage would have been higher than it was. However, even in the lockdown, the tool is still being accessed and used, though to a smaller extent. This suggests that those who had registered for the tool before the lockdown have continued to use it off campus, likely having formed the habit of use.

## Conclusions and recommendations

Data suggests that though UpToDate is used in a variety of ways by a variety of user types at the university, usage remains relatively low considering the total number of students, faculty, and potential searches. Therefore, there is a need for continued advocacy and capacity building for the various users of the tool and others like librarians, who promote its uptake and usage. As for the librarians, who promote the uptake and usage of the tool, there is need for further capacity building and awareness. The usage trend, however, suggests that, irrespective of what method of access used to access the tool, users had started embracing the tool before the COVID-19 pandemic and closure of institution. Continued use in such difficult times suggests that there is potential for increased and more consistent use in the future.

Increased capacity building and promotion is expected to go a long way in increasing usage of evidence-based digital tools. The Better Evidence for Champions program, launched in August 2020, in which local librarians, faculty members, and ICT professionals are trained to aid in promoting uptake, is one possibility for increasing uptake via trained local advocates. As bi-monthly data collection will continue, further analysis after the launch of the Champions program will be conducted. This project and other similar efforts that aim to promote the use of evidence in clinical care will be critical to improving the quality of care and health outcomes for patients for decades to come.

## Limitations of the study

The demographic characteristics of the respondents were not captured because of the nature of the study. The data was remotely captured based on log ins in the system, and this could not allow for the capture of data, other than the number of log ins and the topics viewed.
